# Links between transactional sex and HIV/STI-risk and substance use among a large sample of European men who have sex with men

**DOI:** 10.1186/s12879-019-4326-3

**Published:** 2019-08-05

**Authors:** Rigmor C. Berg, Peter Weatherburn, Ulrich Marcus, Axel J. Schmidt

**Affiliations:** 1 Norwegian Institute of Public Health, PO Box 4404, Nydalen, N-0403 Oslo, Norway; 2 University of Tromso, Hansine Hansens veg 18, N-9019 Tromso, Norway; 3 Sigma Research, Department of Public Health, Environments and Society, London School of Hygiene and Tropical Medicine, 15-17 Tavistock Place, London, WC1H 9SH England; 4 Department of Infectious Diseases Epidemiology, Robert Koch-Institute, Berlin, Germany

**Keywords:** Men who have sex with men, Transactional sex, HIV, Sexually transmitted infections, Drug use, Europe

## Abstract

**Background:**

In Europe, the highest proportion of HIV diagnoses are in gay men and other men who have sex with men (MSM). Globally, HIV prevalence is particularly high among males who report selling sex, but rates among men who buy sex from other men are less clear. This study analyzed the association of transactional sex (TS) and HIV diagnosis, sexually transmitted infection (STI) diagnoses, and various drug use; and examined the variations in TS by payment direction.

**Methods:**

We conducted a cross-sectional, non-randomized, observational study. This European MSM Internet Survey recruited MSM from 38 European countries. For descriptive purposes we stratified according to TS behavior (frequently selling sex, frequently buying sex, neither frequently selling nor buying sex in the previous 12 months), and we constructed separate multivariable logistic regression models to investigate whether engaging in TS accounted for some of the HIV- and STI diagnoses and drug use in this population.

**Results:**

Of almost 161,000 sexually active MSM, 12.2% engaged in TS. The multivariable logistic regression results showed that relative to not frequently engaging in TS, frequently selling sex was independently associated with a higher odds of reporting diagnosed HIV (ever, adjusted odds ratio [aOR] 1.60, confidence interval [CI] 95% 1.39 to 1.85), bacterial STIs (past 12 months, aOR 1.75 CI 95% 1.54 to 2.00), using heroin or crack cocaine or injecting drugs (aOR 3.17, CI 95% 2.70 to 3.73), and using benzodiazepines (aOR 2.13, CI 95% 1.88 to 2.41). Compared to men not engaging in frequent TS, frequently buying sex was associated with a higher odds of using benzodiazepines (aOR 2.13, CI 95% 1.88 to 2.41).

**Conclusions:**

MSM who frequently sell sex suffer greater sexual- and substance use risks than other MSM, but both men who frequently sell and those who buy sex are more likely to use benzodiazepines. MSM who sell sex to other men constitute an important at-risk population who must be offered targeted health services.

## Background

In Europe, the highest proportion of HIV diagnoses are in gay men and other men who have sex with men (MSM), with sex between men accounting for 40% of all new HIV diagnoses in 2016 [1]. HIV prevalence is particularly high among men who report selling sex [2]. Selling and buying sex, collectively referred to as transactional sex (TS), is generally defined as the trading of sex for material goods like money, drugs, or shelter. This includes informal bartering by individuals whose primary income is not derived from TS [3–5]. Among gay, bisexual and other MSM, recent studies indicate that in post-industrialized countries, in the last year 4.5–7.0% have sold [4, 6–8] and 6.0–17.1% have paid for sex with another man [4, 6, 8].

A 2014 literature review of 66 studies and almost 32,000 men found that MSM who sell sex are disproportionately affected by HIV, with more than 20 times the prevalence of HIV infection relative to the general male population. In Europe, nine small studies indicated the HIV prevalence rate was 12.2% among MSM who sell sex [2]. However, there was no comparison of MSM who sell sex with MSM who do not sell sex and it was not clear whether selling sex *itself* represents an increased risk for HIV acquisition. For example, it is known that STIs increase the risk of HIV transmission and acquisition [9]. While a handful of studies report varying STI rates among men who sell sex [10–13], it is unclear whether rates are higher among these men compared to other MSM. Data from U.S. MSM, collected in 2008, showed that both MSM who sold and bought sex in the last 60 days reported higher STI rates than other MSM, but transactional sex was not associated with HIV diagnosis [4].

Similarly, MSM who sell sex seem to be more sexually adventurous in general, with several studies [8, 14] indicating that these men are more sexually active, sensation-seeking, and engage in more condomless sex than other MSM. Importantly, use of various recreational drugs is associated with sexual disinhibition [15, 16]. However, there is limited and conflicting evidence as to whether selling sex is associated with concurrent recreational drug use [7, 8, 10, 14]. Benzodiazepines are anxiolytic drugs commonly prescribed to people living with HIV to decrease social inhibition and anxiety [17]. While to our knowledge their use among men who engage in TS hitherto is unexamined, there is some early data showing that selling sex is associated with mental health problems often treated with sedatives and tranquilizers, including elevated levels of emotional distress [18], psychological distress [19], and other indications of increased mental health problems [11, 20, 21].

There is a scarcity of studies of men buying sex from men, and many of them take the form of descriptive typologies. The comparative studies that have been conducted suggest that MSM who buy sex are more likely than other MSM to be older [4, 6, 8, 22], have university level education [6, 8], steady employment [6], lower rates of syphilis [14], higher levels of alcohol use and more frequent stimulant use [22], and to be more sexually adventurous, and rate themselves as relatively less attractive [8]. There is also some evidence that MSM who buy sex from men are mostly single, HIV-negative, and identify as gay [23].

MSM who sell sex and MSM who buy sex are socio-demographically different and they may also have different health profiles. In order to understand the contribution of TS to HIV risk and other risks, it is important to try to disentangle the relative contribution of other factors. This analysis therefore investigated health related outcomes of selling and buying sex, by payment direction, in an effort to delineate factors that may be used to inform health services for these populations of MSM. The objectives of the study were to explore the relationship between TS and other behavioral health risks; analyze the association of TS and HIV diagnosis, STI diagnoses, and drug use, controlling for other risks; and examine the variations in TS by payment direction. Based on previous research (see above) we hypothesized that selling, but not buying sex would be positively associated with diagnosed HIV infection and self-reported STIs, while both buying and selling sex would be positively associated with drug use.

## Methods

### Procedures

We used data from the European MSM Internet Survey (EMIS-2010), a cross-sectional study conducted simultaneously in 38 countries in 2010 with the objective of identifying prevention needs commonly unmet across diverse groups of MSM. Promotion of the study was through more than 230 social media and dating websites for gay, bisexual and other MSM. We collected data through an anonymous (neither names nor Internet protocol addresses were collected), self-administered survey, accessible online from June 6 to August 31, 2010. It was available in 25 languages and the typical completion time was 20 min (auto-captured by the survey software). Participants were required to indicate that they understood the purpose of the study and consented to take part. They received no recompense. We give detailed descriptions of the methods of EMIS-2010, including minor variations in methods among the 38 participating countries, elsewhere [24, 25].

### Participants

Eligibility criteria were residing in Europe and being a man who had sex with men and/or felt attracted to men. Participants also had to be legally of age to have consensual sex with men in their country of residence and consent to participate in the study.

### Measures and statistical analysis

The survey was developed over several rounds of testing (see [24, 25]). The final version included mainly closed-ended questions, with answer options being largely recency scale, Likert scale, and binary (e.g., yes/no). With respect to the present analysis, all participants who reported any sexual contact with at least one man in the previous 12 months were asked how frequently they had “been paid by a man to have sex”, and “paid a man for having sex” with them in their country of residence (the survey auto-displayed the country name that was selected previously as the respondent’s country of residence). The frequency scale was: Not at all, 1–2 times, 3–10 times, 11–50 times, More than 50 times. To be consistent with a previous analysis [6], we operationalized frequently selling sex as having been paid by a man to have sex 11 or more times in the previous 12 months, and frequently buying sex as having paid a man to have sex three or more times in the previous 12 months.

With respect to the criterion variables, in accordance with our aim and to fill gaps in the literature, we examined four health outcomes. We examined (i) having (ever) been diagnosed with HIV, and (ii) having been diagnosed with a bacterial STI in the past 12 months, including syphilis, gonorrhea, Chlamydia. We also assessed (iii) use of illegal drugs with a high risk of physical harm: heroin, crack cocaine, and injection of any recreational drugs (other than anabolic steroids and medicines). Lastly, (iv) we assessed use of benzodiazepines (sedatives and tranquilizers), which are physically and psychologically addictive. The recall period for all behaviors was the last 12 months.

We ran descriptive analyses and assessed the difference between groups to characterize the frequency with which participants reported a range of health-related behaviors and experiences. For descriptive purposes, we stratified according to TS behavior (frequently selling sex, frequently buying sex, neither frequently selling nor buying sex in the previous 12 months).

To examine the hypothesized influence of TS behavior on health outcomes, we first conducted separate logistic regression models for each of the four outcomes. Next, we constructed separate multivariable logistic regression models for each outcome to determine the independent influence of TS behavior. Covariates were age, education, and number of partners because of their documented influence (see e.g. [26, 27]). We show the odds ratios (ORs) and the adjusted OR (aORs) and 95% confidence intervals (95% CI) for each variable in the models. We used Statistical Package for the Social Sciences Version 24 software to perform all analyses, which were two-tailed with significance set at the 1% level.

## Results

Of the 184,469 submitted survey responses, 174,209 (94.4%) passed the internal data validity checks and made up the final sample (age 13–89 years, mean 34.1, standard deviation 11.3, reflects that age of homosexual consent varies from 13 to 18 years across Europe). Potential sampling biases and representativeness of the sample have been checked with only minor sampling biases identified. [28–30].

As Table 1 shows, close to 161,000 MSM reported any sexual contact with at least one man in the previous 12 months. Frequent sellers made up 1.0% (*n* = 1650) and frequent buyers made up 3.0% (*n* = 4910) of respondents who answered the questions about being paid and paying for sex in the country in which they resided. Altogether, while 12.2% engaged in TS (4.5% reported selling, 7.0% reported buying, 0.7% reported both buying and selling), most of the MSM who engaged in TS did so 1 to 2 times in the past year. Socio-demographically, compared to men who frequently paid for sex and men who neither frequently sold nor paid for sex, a higher proportion of MSM who reported selling sex 11 or more times in the previous 12 months were younger than 39 years old, unemployed, lived in a large city, and were born in another country. Conversely, MSM who reported buying sex three or more times in the past year were generally over 40 years old, single or in a relationship with a woman, had higher education, and fewer were unemployed.

**Table 1:** Sociodemographic characteristics of the EMIS-2010 sample, total and grouped by transactional sex behavior (*N* = 160,719)

	Frequent selling	Frequent buying	Not frequent TS	Total sample
	*n* = 1650	*n* = 4910	*n* = 154,159	*n* = 160,719
Age				
<20 yrs	182 (11.1)	29 (0.6)	8400 (5.5)	8599 (5.4)
20–24 yrs	457 (27.6)	121 (2.5)	26,538 (17.1)	27,097 (16.8)
25–29 yrs	375 (22.7)	297 (6.0)	27,875 (18.1)	28,530 (17.8)
30–39 yrs	420 (25.5)	1242 (25.3)	44,978 (29.2)	46,664 (29.0)
≥40 yrs	216 (13.1)	3221 (65.6)	46,368 (30.1)	49,829 (31.0)
Education ^1^				
High (ISCED 5–6)	568 (34.9)	2837 (58.0)	77,744 (50.6)	81,142 (50.7)
Mid (ISCED 3–4)	763 (46.9)	1711 (35.0)	63,900 (41.6)	66,366 (41.5)
Low (ISCED 1–2)	296 (18.2)	340 (7.0)	11,850 (7.8)	12,508 (7.8)
Sexual orientation				
Gay or homosexual	1155 (70.3)	3674 (75.1)	120,232 (78.1)	125,028 (78.1)
Bisexual	289 (17.6)	843 (17.2)	21,539 (14.0)	22,394 (14.0)
Straight or heterosexual	24 (1.5)	28 (0.6)	824 (0.5)	873 (0.5)
Other or don’t use a term	176 (10.7)	347 (7.1)	11,225 (7.4)	11,774 (7.4)
Relationship status				
Steady relationship with man/men	657 (39.9)	1592 (32.4)	64,502 (41.9)	66,808 (41.7)
Steady relationship with woman/women	83 (5.0)	464 (9.5)	8337 (5.4)	8831 (5.5)
Steady relationship with man and woman	21 (1.3)	43 (0.9)	790 (0.5)	854 (0.5)
Single	886 (53.8)	2811 (57.2)	80,225 (52.1)	83,901 (52.3)
Occupation				
Employed full- or part time	984 (60.1)	3063 (62.6)	111,168 (72.3)	116,212 (72.5)
Unemployed	226 (13.8)	174 (3.5)	9107 (5.9)	9498 (5.9)
Self-employed	358 (21.9)	1017 (20.8)	17,766 (11.6)	19,135 (11.9)
Student	262 (16.0)	86 (1.8)	23,303 (15.2)	23,681 (14.8)
Retired	22 (1.3)	336 (6.9)	3514 (2.3)	3866 (2.4)
Other	142 (8.7)	216 (4.4)	6600 (4.3)	6952 (4.3)
Closeted about sexual identity				
Out to no-one or very few	381 (23.4)	1784 (36.5)	44,733 (29.2)	46,908 (29.3)
Out to more than a few	1247 (76.6)	3097 (63.5)	108,697 (70.8)	113,031 (70.7)
Settlement size				
≥500,000	975 (61.3)	2549 (53.9)	69,393 (46.1)	72,912 (46.5)
<500,000	616 (38.7)	2176 (46.1)	81,091 (53.9)	83,891 (53.5)
Region of residence				
WHO “Western Europe”	1404 (85.1)	4320 (88.0)	132,315 (85.8)	138,024 (85.9)
WHO “Central Europe”	163 (9.9)	315 (6.4)	13,968 (9.1)	14,469 (9.0)
WHO “Eastern Europe”	83 (5.0)	274 (5.6)	7876 (5.1)	8226 (5.1)
Born in country of residence				
Yes	1189 (73.6)	3988 (84.5)	129,526 (86.0)	134,773 (85.9)
No, born in another country	427 (26.4)	732 (15.5)	21,121 (14.0)	21,198 (13.6)

Legend: *ISCED* International Classification of Education (1997), where ISCED 1 is primary education and ISCED 6 is the second stage of tertiary education (e.g.,Ph.D.). *TS* Transactional sex. Region of residence were grouped into the World Health Organization European sub-regions

Table 2 shows that a higher proportion of men reporting frequently selling sex were diagnosed with HIV (ever) or in the past 12 months having syphilis, gonorrhea or Chlamydia, used stimulant drugs (Methyl​enedioxy​methamphetamine [MDMA]/ecstasy, amphetamines/speed, crystal methamphetamine, mephedrone, gamma-hydroxybutyrate/butyrolactone [GHB/GBL], ketamine), engaged in condomless anal intercourse with a non-steady male partner, and debuted sexually at age 17 or younger. They were also more likely to have good knowledge about HIV and HIV testing, test for HIV and STIs in the past 12 months, and ever have used post-exposure prophylaxis. Compared to men not frequently engaging in TS, the health-related profile of men frequently buying sex was characterized by sometimes feeling lonely, consuming alcohol, and using sedatives or tranquilizers.

**Table 2:** Health-related characteristics of the EMIS-2010 sample, by transactional sex behavior in the last 12 months

Variables	Frequent selling	Frequent buying	Not frequent TS
	n (%)	n (%)	n (%)
HIV-positive diagnosis (ever)	271 (16.5)	539 (11.2)	12,011 (7.8)
Tested for HIV	796 (55.2)	1501 (34.2)	56,542 (39.1)
STI diagnosis (syphilis, gonorrhoea, Chlamydia)	338 (20.7)	358 (7.5)	9737 (6.4)
Tested for STIs	894 (57.6)	1537 (34.1)	49,697 (34.0)
Good knowledge about HIV and HIV testing	1076 (65.3)	2896 (60.4)	89,415 (58.0)
Age ≤ 17 at first sexual experience with men	1194 (73.6)	2388 (50.9)	74,920 (49.6)
Engaged in CAI with non-steady male partner	915 (64.3)	1780 (41.8)	43,294 (38.4)
Engaged in CVI intercourse with any woman	208 (59.6)	372 (64.8)	10,381 (63.2)
Used Post-Exposure Prophylaxis (PEP) ever	94 (5.8)	159 (3.4)	3731 (2.5)
Consumed tobacco products in the last 24 h	872 (53.4)	1670 (35.1)	61,819 (40.3)
Consumed alcohol in the last 24 h	620 (37.9)	2151 (45.0)	60,922 (39.6)
Used stimulant drugs^a^	644 (39.4)	651 (13.7)	20,346 (13.3)
Used sedatives or tranquilizers	344 (21.2)	667 (14.1)	15,013 (9.8)
Used heroin, crack cocaine and/or has injected any recreational drugs other than steroids	210 (13.6)	154 (3.3)	3593 (2.4)
Sometimes feel lonely	938 (57.0)	2828 (59.2)	83,172 (54.1)

Note: Time of recall is past 12 months unless otherwise stated. *CAI* Condomless anal intercourse, *CVI* Condomless vaginal intercourse, *TS* Transactional sex. ^a^Stimulant drugs = MDMA/ecstasy, amphetamines/speed, crystal methamphetamine, mephedrone, GHB/GBL, ketamine, cocaine. Test for difference (*p*) < 0.001 for all variables except engaged in condomless intercourse with a woman (*p* = 0.276)

**Fig. 1:**
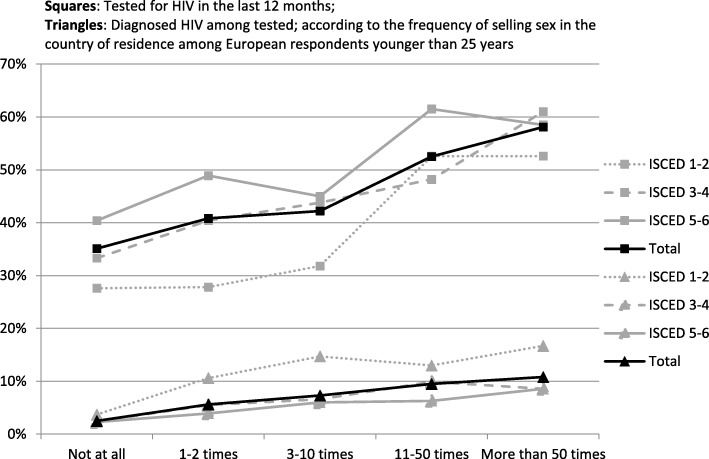
HIV testing and HIV diagnosis among MSM by frequency of selling sex, stratified by educational levels (ISCED 1 through 6)

In Table 3, we show the results of the multivariable logistic regression models. With respect to HIV diagnosis, in the context of the other variables, this outcome was significantly more likely among MSM frequent selling sex (aOR 1.60, CI 95% 1.39 to 1.85) and less likely among men who reported frequent buying sex (aOR 0.85, CI 95% 0.77 to 0.93). The odds of being diagnosed with HIV increased with age (over 25 years aOR 4.83 to 9.46), number of sex partners (two or more partners aOR 1.20 to 5.76), and lower education (aOR 1.28 to 1.58). Figure 1 illustrates the association between the frequency of selling sex and HIV-testing and diagnosis, stratified by education.

**Table 3:** Multivariable logistic regression analysis of influence of transactional sex on HIV- and STI diagnosis, and drug use (injection drug use, benzodiazepines)

Variables	HIV diagnosis (ever)		STI diagnosis^1^		Injection drug use^1^		Use of benzodiazepines^1^	
	OR (95% CI)	aOR (95% CI)	OR	aOR	OR	aOR	OR	aOR
Transactional sex								
Neither frequent buying nor selling sex	*Ref*		*Ref*		*Ref*		*Ref*	
Frequent buying sex	1.50 (1.37–1.64)	0.85 (0.77–0.93)	1.20 (1.07–1.34)	0.87 (0.78–0.97)	1.40 (1.19–1.65)	1.20 (1.01–1.42)	1.50 (1.38–1.66)	1.37 (1.26–1.50)
Frequent selling sex	2.33 (2.04–2.66)	1.60 (1.39–1.85)	3.86 (3.41–4.35)	1.75 (1.54–2.00)	6.40 (5.51–7.42)	3.17 (2.70–3.73)	2.47 (2.19–2.78)	2.13 (1.88–2.41)
Age: < 25	*Ref*		*Ref*		*Ref*		*Ref*	
25–39	5.34 (4.90–5.83)	4.83 (4.42–5.29)	1.68 (1.59–1.78)	1.31 (1.23–1.39)	1.26 (1.12–1-40)	1.00 (0.91–1.08)	1.27 (1.22–1.32)	1.17 (1.12–1.23)
40 +	11.27 (10.27–12.20)	9.46 (8.65–10.35)	1.47 (1.38–1.56)	1.06 (0.99–1.13)	1.12 (1.05–1.20)	0.83 (0.76–0.92)	1.32 (1.26–1.39)	1.17 (1.11–1.23)
Number of sex partners^1^: 1	*Ref*		*Ref*		*Ref*		*Ref*	
2–10 sex partners	1.30 (1.23–1.37)	1.20 (1.13–1.27)	2.40 (2.23–2.58)	2.06 (1.91–2.22)	0.34 (0.31–0.37)	1.55 (1.40–1.72)	1.07 (1.03–1.11)	1.06 (1.02–1.11)
11–50 sex partners	2.89 (2.73–3.04)	2.39 (2.25–2.53)	6.35 (5.91–6.83)	5.32 (4.93–5.73)	0.53 (0.49–0.57)	2.95 (2.65–3.28)	1.33 (1.27–1.40)	1.26 (1.20–1.33)
50 + partners	7.51 (7.01–8.05)	5.76 (5.39–6.21)	14.86 (13.64–16.19)	11.94 (10.90–13.07)	2.37 (2.14–2.63)	6.10 (5.34–6.97)	1.85 (1.72–2.00)	1.63 (1.51–1.77)
Education: High	*Ref*		*Ref*		*Ref*		*Ref*	
Mid	0.98 (0.94–1.02)	1.28 (1.23–1.33)	0.83 (1.78–0.90)	0.98 (0.94–1.02)	1.12 (1.05–1.20)	1.22 (1.14–1.31)	0.83 (0.80–0.86)	0.88 (0.84–0.91)
Low	1.34 (1.26–1.42)	1.58 (1.48–1.69)	0.82 (0.79–0.86)	1.10 (0.93–1.09)	1.26 (1.13–1.40)	1.37 (1.22–1.54)	1.04 (0.98–1.10)	1.08 (1.01–1.14)

Legend: 1 = In the past 12 months. For explanation of education, see Table 1. STI diagnoses include syphilis, gonorrhea, Chlamydia. Injection drug use includes any use of heroin or crack cocaine or injection of any recreational drugs other than steroids

Similarly, the final multivariable model for reporting a bacterial STI showed that engaging in TS remained significantly associated with STIs. Relative to men who reported neither frequently buying nor selling sex, STIs were statistically more likely among men selling sex (aOR 1.75, CI 95% 1.54 to 2.00) and less likely among men buying sex (aOR 0.87, CI 95% 0.78 to 0.97). The odds of reporting being diagnosed with syphilis, gonorrhea or Chlamydia was statistically more likely among those 25–39 years (aOR 1.31, CI 95% 1.23 to 1.39) and increased with number of sex partners (two or more partners aOR 2.06 to 11.94). The third model, for heroin, crack cocaine, and injection drug use, showed that both frequently selling sex (aOR 3.17, CI 95% 2.70 to 3.73) and buying sex (aOR 1.20, CI 95% 1.01 to 1.42) were significant predictors. Being over 40 years (aOR 0.83, CI 95% 0.763 to 0.92), reporting a higher number of sex partners (two or more partners aOR 1.55 to 6.10) and having lower education (aOR 1.22 to 1.37) were also associated with injection drug use. As shown in the fourth and final model, relative to men who reported no TS, both men who frequently sold (aOR 2.13, CI 95% 1.88 to 2.41) and frequently bought sex (aOR 1.37, CI 95% 1.26 to 1.50) were more likely to use benzodiazepines (sedatives or tranquilizers). Also age (aOR 1.17), number of sex partners (aOR 1.06 to 1.63) and education (aOR 0.88 to 1.08) were associated with use of benzodiazepines.

## Discussion

Few studies have examined health correlates of buying sex among MSM and even fewer selling and buying sex separately. However, to the extent that MSM who engage in TS have different health risks, these differences may suggest a need for tailored interventions. Indeed, in our study of almost 161,000 sexually active MSM from 38 European countries, we found striking variations in sexual- and substance risks by TS payment direction.

Consistent with our hypotheses, we established that selling sex was independently associated with a higher likelihood of being HIV positive, having STIs, injecting drugs, and using benzodiazepines. More than twice as many MSM who frequently sold sex compared to men who neither sold nor bought sex frequently were HIV-positive. The 16.5% rate of HIV diagnosis among men who sell sex is somewhat higher than that found in earlier European studies [2], indicating a possible rise in HIV among this sub-group. The correspondingly high rate of STIs, with more than one in five men stating they were diagnosed with syphilis, gonorrhea, or Chlamydia in the past year, was another sexual transmission risk independently correlated with selling sex. This finding strengthens results from similar, recent studies conducted in China and Ecuador [12–14], and in part, an older study from the U.S. [4].

With respect to substance use, a behavior linked with sexual disinhibition and increased risk of HIV acquisition [16], 39.4% of men in our sample who sold sex reported using stimulant drugs – in contrast to 13% among other MSM – and 13.6% used heroin, crack cocaine or injected drugs. In fact, men who sold sex were over three times more likely to use heroin, crack cocaine or inject drugs, which corroborates results from earlier studies in the U.S. [4, 22, 31], and adds to the limited knowledge base from Europe [10, 32]. Consuming legal and illegal substances may be a mechanism used by MSM to cope with psychosocial stress in general [11] and benzodiazepines use may be used to deal with social inhibition and anxiety in particular. Consistent with our hypothesis, after adjusting for other factors, both frequently being paid for and paying for sex were associated with a higher odds of using sedatives and tranquilizers. It is possible that trading sex, irrespective of payment direction, elevates men’s levels of emotional distress, which they manage with benzodiazepines. Our study is one of the first to examine their use among MSM who trade sex, which prevents comparison with other studies. Yet, related research suggests that men who sell sex to other men may be particularly at risk given their more vulnerable socio-demographic status [4, 6, 8, 33], reduced mental health [11, 18–21], and HIV-positivity [2]. However, it is possible that the elevated use of tranquilizers among men who frequently sell sex may be a ‘side effect’ of elevated use of stimulant drugs, and that men who suffer from elevated distress are more likely to engage in TS.

The health- and behavioral characteristics of MSM who reported frequently buying sex in the past year differed from those of both MSM frequently selling sex and those neither buying nor selling. In most respects, buyers reported less risk than men selling sex but more than men reporting no TS. It is worth noting that, relative to MSM sellers, buyers reported more bisexual activity, lower engagement in condomless anal sex, but a higher proportion reported sometimes feeling lonely, and not testing for HIV and STIs. After adjusting for number of partners, age, and education, buying sex was only borderline associated with having HIV- and STI diagnoses, and injection drug use, but, as discussed above, buying sex was significantly associated with higher likelihood of using benzodiazepines. Related, a higher proportion consumed alcohol, which also lowers social inhibition and anxiety. Our findings on bisexual behavior, alcohol use, and rates of HIV and STIs are in line with past research [14, 22, 23], but the health profile and risks of men who buy sex from other men require more study. While research into many aspects of TS between men is important, it seems especially relevant to examine also the relationship between TS and mental health and substance use, preferably in longitudinal studies to enable more causal conclusions.

Our study has several strengths and limitations. First, this is the largest study on MSM in general and on MSM who engage in TS in particular, with a good range of MSM milieus. We used valid measures, multivariable analyses, a broad definition of TS that included informal sex trading, and conducted one of the first analyses to examine aspects of health associated with TS by payment direction. Yet, we have not examined causation and this is a non-random sample that cannot be assumed to be representative of all MSM in Europe. As most surveys, EMIS may be biased towards middle-class participants, such that the differences observed here may be more pronounced in lower socio-demographic strata. All data were self-reported and limitations such as recall bias, social desirability bias, and measurement bias may affect the findings. Lastly, we assessed transactional sex in the respondents’ country of residence and have not captured transactional sex that may have occurred elsewhere.

## Conclusions

The present study’s findings of strong relationships between TS and health risks and the striking variations in risk by TS payment direction have important implications. First, the finding that selling sex, but not buying sex, represents an elevated risk for HIV- and STI acquisition confirm that men who sell sex to other men constitute an important at-risk population in the European HIV/STI epidemic, who are in need of targeted interventions. Second, the clustering of risks for these men, including injection drug use and other hard drugs, suggests they must be offered comprehensive sexual- and substance risk-reduction services as well as health services that prevent escalation of their vulnerability. Our findings identify that priority groups of men who sell sex include men who are younger and have lower socio-economic status, including being a migrant. Moreover, given this group’s uptake of clinical services and evidence that clinic-visits provide opportunities for risk-reduction counselling, STI management and HIV testing [12], sexual health clinics emerge as a promising location for reaching such men. Finally, in concert with data showing that TS is linked with mental health problems [11, 18–21], our finding that engagement with TS is strongly associated with use of benzodiazepines point to a need for mental health evaluation and possibly treatment for men who trade sex.

## Data Availability

The dataset used and/or analysed during the current study are available from the corresponding author on reasonable request.

## Abbreviations

aOR: adjusted odds ratio
CI: Confidence interval
EMIS-2010: European MSM Internet Survey, conducted in 2010
GHB/GBL: Gamma-hydroxybutyrate/butyrolactone
ISCED: International Classification of Education
MDMA: Methyl​enedioxy​methamphetamine
MSM: Men who have sex with men
OR: Odds ratio
STI: Sexually transmitted infections
TS: Transactional sex
